# Correction: Rosavin improves insulin resistance and alleviates hepatic and kidney damage *via* modulating the cGAS-STING pathway and autophagy signaling in HFD/STZ-induced T2DM animals

**DOI:** 10.1039/d4md90030h

**Published:** 2024-08-02

**Authors:** Hebatallah S. Ali, Hiba S. Al-Amodi, Shaimaa Hamady, Marian M. S. Roushdy, Amany Helmy Hasanin, Ghada Ellithy, Rasha A. Elmansy, Hagir H. T. Ahmed, Enshrah M. E. Ahmed, Doaa M. A. Elzoghby, Hala F. M. Kamel, Ghida Hassan, Hind A. ELsawi, Laila M. Farid, Mariam B. Abouelkhair, Eman K. Habib, Mohamed Esawie, Heba Fikry, Lobna A. Saleh, Marwa Matboli

**Affiliations:** a Medical Biochemistry and Molecular Biology Department, Faculty of Medicine, Ain Shams University Cairo 11566 Egypt DrMarwa_Matboly@med.asu.edu.eg; b Biochemistry Department, Faculty of Medicine, Umm Al-Qura University Makkah 21955 Saudi Arabia; c Biochemistry Department, Faculty of Science, Ain Shams University Cairo Egypt; d Clinical Pharmacology Department, Faculty of Medicine, Ain Shams University Cairo Egypt; e Anatomy Unit, Department of Basic Medical Sciences, Unaizah College of Medicine and Medical Sciences, Qassim University Buraydah Saudi Arabia; f Anatomy Unit, Department of Basic Medical Sciences, College of Medicine and Medical Sciences, AlNeelain University Sudan; g Pathology Unit, Department of Basic Medical Sciences, College of Medicine and Medical Sciences, Gassim University Saudi Arabia; h Clinical Pathology, Faculty of Medicine, Ain Shams University Egypt; i Physiology Department, Faculty of Medicine, Ain Shams University Egypt; j Department of Internal Medicine, Badr University in Cairo Badr City Egypt; k Pathology Department Faculty of Medicine, Ain Shams University Egypt; l Department of Anatomy and Cell Biology, Faculty of Medicine, Ain Shams University Egypt; m Department of Anatomy and Cell Biology, Faculty of Medicine, Galala University Egypt; n Department of Histology, Faculty of Medicine, Ain Shams University Cairo Egypt; o Department of Clinical Pharmacology, Faculty of Medicine, Ain Shams University Cairo Egypt

## Abstract

Correction for ‘Rosavin improves insulin resistance and alleviates hepatic and kidney damage *via* modulating the cGAS-STING pathway and autophagy signaling in HFD/STZ-induced T2DM animals’ by Hebatallah S. Ali *et al.*, *RSC Med. Chem.*, 2024, **15**, 2098–2113, https://doi.org/10.1039/D4MD00023D.

The authors regret the misspelling of the surname of one of authors, Mohamed Esawie, in the original manuscript. The corrected list of authors for this paper is shown above.

The Royal Society of Chemistry apologises for these errors and any consequent inconvenience to authors and readers.

